# Executive function in schizophrenia and autism in adults shares common components separating high and low performance groups

**DOI:** 10.3389/fpsyt.2024.1381526

**Published:** 2024-04-18

**Authors:** Sofia Morais, Otília C. d’Almeida, Salomé Caldeira, Sofia Meneses, Graça Areias, Vanessa Girão, Catarina Bettencourt, Daniela Jardim Pereira, António Macedo, Miguel Castelo-Branco

**Affiliations:** ^1^ Psychiatry Department, Coimbra Hospital and University Centre, Coimbra, Portugal; ^2^ Faculty of Medicine, University of Coimbra, Coimbra, Portugal; ^3^ Coimbra Institute for Biomedical Imaging and Translational Research (CIBIT), University of Coimbra, Coimbra, Portugal; ^4^ Institute of Nuclear Sciences Applied to Health (ICNAS), University of Coimbra, Coimbra, Portugal; ^5^ Psychology Department, Coimbra Hospital and University Centre, Coimbra, Portugal; ^6^ Faculty of Psychological and Education Sciences, University of Coimbra, Coimbra, Portugal; ^7^ Centre for Research in Neuropsychology and Cognitive and Behavioral Intervention (CINEICC), Faculty of Psychological and Education Sciences, University of Coimbra, Coimbra, Portugal; ^8^ Neurorradiology Functional Area, Imaging Department, Coimbra Hospital and University Center, Coimbra, Portugal; ^9^ Institute of Psychological Medicine, Faculty of Medicine, University of Coimbra, Coimbra, Portugal

**Keywords:** executive function, schizophrenia, autism spectrum disorder, neuropsychology, stroop

## Abstract

The profile of executive function (EF) in adults with Schizophrenia (SCZ) and autism spectrum disorder (ASD) remains unclear. This study aims to ascertain if distinct EF patterns can be identified between each clinical condition by comparing the neuropsychological profile of adults with SCZ and ASD, for whom the differential diagnosis is still highly challenging. Forty-five individuals (15 SCZ, 15 ASD, 15 controls) matched for age, sex, education level, and handedness underwent intelligence evaluation and neuropsychological testing for working memory, inhibition, planning and set-shifting, and verbal fluency subdomains. Principal component analysis (2D-PCA) using variables representing 4 domains was employed to identify patterns in neuropsychological profiles. The ASD group had lower scores on the Digits Forward subtest compared to the SCZ group (7.2 ± 2.1 vs. 9.3 ± 1.9, *p* = 0.003; Cohen’s *d*: 1.05). ASD also performed significantly worse on the Stroop Word Test compared to the control group (77.7± 17.9 vs. 98.0 ± 12.7, *p* = 0.009; Cohen’s *d*: 1.31). No significant differences were observed between ASD and SCZ on other EF measures. The larger contributors for the dimensions in 2D-PCA were the Digits Forward subtest and Stroop Word Test. Still, there was substantial overlap between the clinical groups. This study suggests a high degree of similarity of EF between SCZ and ASD. Through four EF measures, the discrimination of low and high-functioning EF groups spanning both diagnostic categories may help to identify the individuals who could better benefit from cognitive rehabilitation strategies.

## Highlights

- Schizophrenia (SCZ) and autism spectrum disorder (ASD) share functional features in executive function subdomains.
- ASD participants were differentially impaired on the *Digits Forward subtest.*
- ASD individuals showed slower response times on the *Stroop Word-Color Test.*
- PCA discriminates between low vs high-functioning groups, helping to stratify for rehabilitation.

## 1 Introduction

The dichotomy of psychoses proposed by Kraepelin has dominated western psychiatry for over a century (1). More recently, in addition to this categorical perspective, in the field of psychoses and specifically in the context of neurodevelopmental disorders, a continuum perspective has also been favored, in which schizophrenia and autism spectrum disorders share some characteristics. There is growing evidence that focuses on the link between schizophrenia (SCZ) and autism spectrum disorder (ASD), with significant overlap in genetic studies (2), neuroimaging data (3), clinical signs and cognitive features (4). However, it is also important to investigate their differences, especially in the cognitive domain, to be able to design tailored cognitive remediation strategies across diagnostic groups.

ASD, typically diagnosed in childhood, is characterized by restricted or repetitive interests or behaviors and impaired social communication, and it tends to have a stable course. SCZ, typically diagnosed in adolescence or adulthood, is characterized by psychotic symptoms (e.g., hallucinations and delusions), but also with declining function. ASD without intellectual disability is often only diagnosed in early adulthood, and these patients can be hard to differentiate from SCZ due to the overlap of common presentations, especially when impaired in social interaction, inability to understand emotions, scarcity of psychotic symptoms, prominence of obsessive-compulsive symptoms, and overlap with SCZ usually diagnosed at this age.

Executive functions (EF) comprise cognitive abilities that enable and drive adaptive, goal-oriented behavior (5). These include: working memory, the ability to generate thought and think flexibly, and to update and monitor information mentally; inhibition, to inhibit what is irrelevant to current goals; set shifting, to modify attention and behavior in response to changing circumstances and demands; and, fluency, the ability to maximize the production of verbal or visual information in a specific time period while avoiding repeating responses (5). Besides, as in other neuropsychological tasks, there is a considerable overlap between the EF components measured within the individual subtests.

EF is known to be fundamental for learning, academic performance, mental health, adaptive and goal-directed behaviors (6). Although scarce, the existing literature suggests that while in SCZ there is a visible decline in EF after psychotic episodes and over time with increasing age (7), in ASD the EF difficulties tend to persist through adulthood (8).

Although an EF impairment was reported in each of these conditions, few studies compared EF in both SCZ and ASD (9–12). In the study of Eack (10), EF was assessed using the Wisconsin Card sorting Test and a cognitive battery (Measurement and Treatment to Improve Cognition in Schizophrenia – MATRICS) consisting of measures of processing speed, attention, working memory, verbal, and visual learning, and problem-solving. They reported that no significant differences were observed between SCZ and ASD in all cognitive domains, and the areas of larger impairment were similar across conditions. These included slowness in processing speed and inability to understand emotions. The main limitations of the study were the heterogeneity of the SCZ group, which included schizophrenia, schizoaffective disorders, and substance consumers, and the cognitive battery was not thoroughly evaluated in adults with ASD. Another study (9) assessed cognitive functions (verbal comprehension, perceptual organization, working memory, and processing speed) using the Wechsler adult intelligence scale-III (WAIS-III). SCZ patients scored significantly lower on processing speed than the ASD and the control group, but no other significant differences were found. However, few EF subdomains were assessed, as EF was only assessed by the subtests of WAIS-III, and there was no assessment of the psychosocial functioning in SCZ and ASD.

Marinopoulou and colleagues (11) studied EF subdomains using the Delis–Kaplan Executive Function System, after the applying WAIS-III. As in De Boer (9), SCZ and ASD scored similarly on EF assessment, except on processing speed, in which SCZ patients scored significantly lower than the ASD group. As a limitation, no neurotypical group was considered. Moreover, ASD group had higher Full-scale Intelligence Quotient (IQ) than SCZ, that was also a significant predictor of the variance of five EF measures in which the clinical groups showed statistically significant differences (11).

More recently, Yon-Hernández (12) found no differences between SCZ and ASD in inhibition performance. SCZ performed poorer than ASD and controls in Updating and Shifting, but their performance improved when there were no time constraints. The results of this study have a few limitations because age was not matched between groups. Overall, the heterogeneity of the neuropsychological tests applied, and the EF subdomains studied, with possible underrepresentation of some cognitive domains, can be strong contributors to the heterogeneity and consistency of results. Principal component analysis (PCA) is a method that allows to identify major cognitive functions by reducing the neuropsychological variables dimensionality to major functional axes. Here we asked if there are distinct executive dysfunction patterns within and between SCZ and ASD, to study their potential application in differential diagnosis and most importantly to help tailor rehabilitation approaches.

In the present study, we applied a battery of neuropsychological tests to evaluate four subdomains of EF: working memory, inhibition, planning and set-shifting, and verbal fluency, in adults with SCZ, ASD, and controls. Our main goal is to compare the functioning of these three groups, matched for age, sex, level of education and handedness. The principal component analysis will allow identifying patterns in neuropsychological profiles by providing additional data reduction while extracting the 2 main functional axes across these disorders.

## 2 Methods

### 2.1 Participants

This study followed a descriptive cross-sectional design with a non-probabilistic sampling approach for participants selection. We included outpatients with schizophrenia (SCZ) and autism spectrum disorder (ASD) from a major university hospital, besides controls, matched for age, sex, education level, and handedness. Intelligence was evaluated using Wechsler Adult Intelligence Scale III (WAIS-III). Inclusion criteria were: (1) DSM-5 criteria for SCZ or ASD (13); (2) age between 18–40; (3) capacity to give consent; (4) handedness through evaluation with the Edinburgh Handedness Inventory (14, 15); (5) clinical stability in the last 6 months prior to enrollment, for the clinical groups. General exclusion criteria were: (1) medical or neurological comorbidity (e.g., epilepsy, head trauma, intellectual disability defined for IQ<80); (2) substance abuse/dependence; (3) contra-indications to magnetic resonance imaging, needed for an MRI protocol outside the scope of this study.

All participants were assessed, by an expert psychologist blinded to the diagnosis, with WAIS-III, Portuguese version (16).

Patients’ clinical assessment included instruments such as the Positive and Negative Syndrome Scale (PANSS) (17) in SCZ group to measure symptoms of SCZ; Autism Diagnostic Observation Schedule Second Edition (ADOS-2) Module 4 (18) in ASD group to confirm the diagnosis, and the Personal and Social Performance Scale (PSP) (19, 20) addressing functioning.

In clinical groups, SCZ and ASD, pharmacological exposure was calculated through defined daily dose – DDD (21), and current antipsychotic exposure was calculated through chlorpromazine equivalents – CPZE (22, 23).

Control individuals were volunteers recruited from the community. A brief interview was performed to exclude personal or family history of psychiatric disorders, in addition to general exclusion criteria. All participants provided written informed consent. The study was approved by the local Ethics Committees of the Faculty of Medicine of the University of Coimbra (ref. CE-043/2020) and Coimbra Hospital and University Centre (ref. CHUC-109-18) in accordance with the Declaration of Helsinki.

### 2.2 Executive function measures

A second expert psychologist, blinded to the performance on the IQ evaluation, assessed four subdomains of EF: working memory, inhibition, planning and set-shifting, verbal fluency. The same battery was administered to all participants in a fixed order.

*Working memory* was examined with the Portuguese versions of *Digit Span task* and *Letter-number sequencing* (16). In the *Digit Span Forward subtest*, the participant must repeat the sequence of numbers stated by the examiner in the same order. It includes eight increasing levels of difficulty from two up to nine-digit sequences. In the *Digit Span Backward subtest*, the participant is asked to repeat the same numbers but in reverse order, i.e., from last to first digit stated. It has seven increasing levels of difficulty from two up to eight-digit sequences. In both Forward and Backward tasks, each level includes two trials of the same length. Participants must correctly reproduce at least one trial at each level to proceed. The task is discontinued when both trials are recalled incorrectly. The accurate responses are calculated by scoring 1 point for each number sequence recalled correctly (forward and backward). In *Letter-number sequencing*, the examinee is presented with 7 triads of a sequence of numbers and letters. After hearing each sequence, the participant is instructed to first recall the numbers in ascending order, and later the letters in alphabetical order. The task is discontinued after the 3 sequences of each triad are recalled incorrectly. The accurate responses are calculated by scoring 1 point for each letter-number sequence recalled correctly.

*Inhibition* was examined with *Stroop Color and Word Test*, Portuguese version (24, 25). The test includes three conditions (Word, Color, and Word-Color). In each condition, examinees are presented with a printed sheet with five columns of 20 stimuli that they must read or name as quickly as possible within 45 seconds. In the first trial (*Stroop Word*), the participant must read the names of the colors that are all written in black on the card (“red”, “green”, or “blue”). In the second trial (*Stroop Color*), the participant is instructed to name the color in which sequences of the letter “X” are printed. In the third trial (*Stroop Word-Color*), the participant must name the color in which the words are written (e.g., the correct response to the word “green” written in red ink would be “red”). The score is the number of correct answers in 45 seconds.

*Planning and set-shifting* was assessed with the *Wisconsin Card Sorting Test-64*, Portuguese version (26, 27) and with the *Trail Making Test* – A and B, Portuguese version (28, 29). The *Wisconsin Card Sorting Test-64* consists of a deck of 64 cards that need to be matched to one of four target cards through trial and error. The participant must match the cards one by one according to a criterion that can be color (red, yellow, blue, or green), shape, or number. After each response, the examiner signals whether the participant matched the card correctly or incorrectly. The matching criterion changes without a warning after 10 consecutive correct matches, i.e., after one successfully completed category. The total number of correct responses, errors, completed categories, and the perseverative responses are considered. Perseverative responses were defined as responses that matched the established perseverated-to principle, i.e., the previous sorting principle that the participant is persisting in. In *Trail Making Test-A* the participant is asked to connect continuously 25 numbers placed randomly on a page in ascending order as fast as possible. *Trail Making Test-B* includes connecting 13 numbers and 12 letters alternately and as quickly as possible without lifting the pencil. If an error is made, it is pointed out by the examiner for correction. The score used was the time, in seconds, required to complete each part.

*Verbal fluency* was assessed with *Production of words under restricted conditions*, which includes *Phonemic Verbal fluency task P-R-M*, Portuguese version (30, 31); and *Semantic Verbal fluency task*, Portuguese version (31, 32). The *Phonemic Verbal fluency task* entails saying as many words, as quickly as possible, beginning with P, R, and M in 60 seconds. Participants cannot use proper nouns or use a stem word with different endings. The *Semantic Verbal fluency task* entails saying as many words within 5 categories (animals, fruits, food, people names, and clothes), as quickly as possible, in 60 seconds, with no restrictions on the first letter or any other characteristics. We calculated the number of correct items generated in 60 seconds.

### 2.3 Clinical assessment

For all participants clinical and demographic data were collected: age, sex, education level, and handedness. Intelligence was evaluated with WAIS-III Portuguese version (16), by an expert psychologist blinded to the diagnose. The four subdomains of EF: working memory, inhibition, planning and set-shifting, verbal fluency, was evaluated by a second expert psychologist, blinded to the performance on the IQ evaluation. The same battery was administered to all participants in a fixed order.

In SCZ group the PANSS (17) was used to measure symptoms of SCZ, by an expert psychiatrist. In ASD group the ADOS-2 Module 4 (18) was used to confirm the diagnosis, by a third expert psychologist blinded to the performance on the IQ evaluation.

In clinical groups SCZ and ASD, PSP (19, 20) was used to address functioning, and pharmacological exposure was calculated, both by an expert psychiatrist.

For controls, medical history was obtained by an interview preceding assessment by an expert psychiatrist.

### 2.4 Statistical analysis

Demographic and clinical data analyses were performed with IBM SPSS Statistics 28.0 (IBM Corporation, New York, EUA). Normality of the data was tested using the Shapiro–Wilk test. When data were normally distributed, parametric tests were used to test differences between groups. If the assumption of normality was not met, non-parametric Kruskal–Wallis H or Mann–Whitney U tests were used to assess between-group differences. Fisher-Freeman-Halton’s exact test was used to assess between-group differences in categorical variables. Comparisons of neuropsychological tests metrics between groups were evaluated with ANCOVA using Performance-IQ as covariate. When appropriate, multiple comparison *post-hoc* tests were conducted with Bonferroni correction. Effect sizes of group comparisons were calculated according to Cohen’s d formula.

To profile the participants based on their EF neuropsychological test performance a two-dimensional unsupervised PCA was conducted using the ‘*FactoMineR’* and ‘*factoextra’* packages in the R Studio software (version 4.2.1). First, a data reduction procedure was applied by directly comparing each neuropsychological test metric between the three groups (ANOVA). Within each EF subdomain, the metric presenting the largest effect size (partial η^2^) was selected. Finally, four variables were included in the 2D-PCA: Digits Forward subtest, Stroop Word Test, Trail Making Test A (time), and Semantic Verbal fluency task. To ensure equal importance of each variable, data were standardized before the PCA. The amount of variation retained by each principal component was based on the eigenvalues.

Study design and data analysis were aligned with the Strengthening the Reporting of Observational Studies in Epidemiology (STROBE) consensus (33).

## 3 Results

### 3.1 Descriptive analysis

Demographic and clinical data are summarized in **Table 1**. All patients with SCZ were on stable antipsychotic medication, predominantly atypical antipsychotics: single second-generation (n = 7), single third-generation (n = 3), a combination of two second-generation (n = 2), or a combination of second and third-generation (n = 3) antipsychotic medication. Within the ASD group, 11 patients were stable without any medication, 1 with a single second-generation antipsychotic medication, and 3 under a combination of antidepressant and second-generation antipsychotics. In the SCZ group, the mean disease duration was 5.6 ± 4.1 years.

**Table 1:** Demographic and clinical data of study groups: schizophrenia (SCZ), autism spectrum disorder (ASD) and controls.

	SCZ (n=15)	ASD (n=15)	Controls (n=15)	Test statistic		*p*-value	Pairwise comparisons^a^
**Sex (male/female)**	15/0	15/0	15/0	–		–	–
**Age (y)**	26.3 ± 5.1	22.9 ± 4.9	25.8 ± 6.8	*F*	1.615	0.211	–
**Education (y)**	13.8 ± 2.6	13.6 ± 2.6	13.7 ± 2.2	*H*	0.180	0.914	–
**Laterality (right/left)**	13/2	15/0	15/0	χ^2^	2.812	0.318	–
**Cigarette smoking** **(pack/year)**	3.1 ± 3.9	0	4.4 ± 5.9	*H*	12.739	0.002	ASD vs. SCZ ^*^ ASD vs. controls ^*^
**Pharmacological exposure, DDD (mg)**	2.2 ± 1.5	0.3 ± 0.5	n.a.	*U*	14.500	<0.001	–
**Antipsychotic exposure, CPZE (mg)**	353.2 ± 270.3	21.1 ± 42.9	n.a.	*U*	2.500	<0.001	–
**Duration of disease (y)**	5.6 ± 4.1	n.a.	n.a.	*-*	*-*	–	–
**FS-IQ**	107.2 ± 11.9	109.4 ± 14.1	116.3 ± 12.4	*H*	4.928	0.085	–
**Verbal-IQ**	111.3 ± 13.4	111.8 ± 14.6	115.3 ± 14.7	*F*	0.355	0.704	–
**Performance-IQ**	101.1 ± 9.6	105.6 ± 15.4	114.1 ± 8.1	*F*	4.891	0.012	SCZ vs. controls ^*^
**Functioning-PSP**	69.5 ± 12.7	67.7 ± 7.0	n.a.	*U*	102.000	0.683	–
**PANSS-P**	11.1 ± 2.7	n.a.	n.a.	*-*	*-*	–	–
**PANSS-N**	16.6 ± 7.1	n.a.	n.a.	*-*	*-*	–	–
**PANSS-GP**	26.8 ± 4.3	n.a.	n.a.	*-*	*-*	–	–
**PANSS**	53.6 ± 11.6	n.a.	n.a.	*-*	*-*	–	–
**ADOS-COM**	n.a.	2.3 ± 1.2	n.a.	*-*	*-*	–	–
**ADOS-SI**	n.a.	8.4 ± 3.3	n.a.	*-*	*-*	–	–
**ADOS-RRB**	n.a	3.7 ± 2.7	n.a	*-*	*-*	–	–
**ADOS-SA**	n.a	10.7 ± 4.1	n.a	*-*	*-*	–	–
**ADOS-2**	n.a	14.4 ± 4.8	n.a	*-*	*-*	–	–

Mean ± Standard deviation.

F = ANOVA test, H = Kruskal–Wallis H test, χ^2^ = Fisher-Freeman-Halton test, U = Mann–Whitney U test.

*p < 0.05.

DDD, defined daily dose; CPZE, chlorpromazine equivalents; FS-IQ, Full-Scale Intelligence Quotient, IQ, Intelligence Quotient; PSP, Personal and Social Performance Scale; PANSS, Positive and Negative Syndrome Scale; P, positive scale; N, negative scale; GP, general psychopathology scale; ADOS, Autism Diagnostic Observation Schedule; COM, Communication; SI, Social Interaction; RRB, Restricted and Repetitive Behavior; SA,Social Affect; n.a., not applicable.

Possible ranges of scores: FS-IQ = [45, 155]; Verbal-IQ = [45, 155]; Performance-IQ = [46, 155]; PSP = [1, 100]; PANSS-P = [7, 49]; PANSS-N = [7, 49]; PANSS-GP = [16, 112]; PANSS = [30, 210]; ADOS-COM = [0, 4]; ADOS-SI = [0, 16]; ADOS-RRB = [0, 10]; ADOS-SA = [0, 20]; ADOS-2 = [0, 30].

Clinical groups (SCZ and ASD) had no relevant demographic differences. The psychopharmacology and antipsychotics exposure, was greater in SCZ patients (*p <*0.001).

Regarding Full-scale Intelligence Quotient evaluation, there were no statistically significant differences between the three groups. However, there was a statistically significant difference in Performance-IQ, greater in controls compared to the SCZ group (*p* = 0.012).

As expected, three patients (n = 2, SCZ; n = 1, ASD) had discrepancies between Verbal-IQ and Performance-IQ, related to the discrepancy in verbal and performance skills that are often described in SCZ (34) and in ASD (35).

Individuals in the ASD group had no exposure to cigarettes. No statistically significant differences were found between SCZ and the control groups in smoking.

### 3.2 Executive functions measures and analysis

The statistics of group performance in executive functions measures and the test statistics corrected for Performance-IQ are summarized in **Table 2**.

**Table 2:** Executive functions measures of study groups: schizophrenia (SCZ), autism spectrum disorder (ASD), and controls.

	SCZ (n=15)	ASD (n=15)	Controls (n=15)	Test statistic corrected for Performance-IQ		*p*-value	Pairwise comparisons^a^
**Digits Forward subtest**	9.3 ± 1.9	7.2 ± 2.1	8.7 ± 1.3	*F*	6.606	0.003	SCZ vs. ASD*
**Digits Backward subtest**	5.5 ± 2.2	6.0 ± 2.1	6.8 ± 2.3	*F*	0.001	0.999	–
**Letter-number sequencing**	9.5 ± 1.7	9.1 ± 2.7	11.6 ± 2.9	*F*	1.924	0.159	–
**Stroop Word Test**	84.1 ± 12.1	77.7 ± 17.9	98.0 ± 12.7	*F*	5.269	0.009	ASD vs. controls*
**Stroop Color Test**	57.9 ± 10.2	60.8 ± 14.5	72.3 ± 10.6	*F*	3.120	0.055	–
**Stroop Word-Color Test**	38.0 ± 9.9	40.0 ± 11.5	47.1 ± 9.3	*F*	1.186	0.316	–
**WCST correct responses**	50.73 ± 7.3	51.6 ± 5.8	52.1 ± 6.8	*F_Q_*	0.031	0.970	–
**WCST total errors**	13.3 ± 7.3	12.4 ± 5.8	11.9 ± 6.8	*F_Q_*	0.031	0.970	–
**WCST perseverative responses**	8.2 ± 5.1	8.4 ± 6.8	8.5 ± 3.0	*F_Q_*	1.660	0.202	–
**WCST categories completed**	4.2 ± 1.3	4.2 ± 1.1	4.3 ± 0.9	*F_Q_*	0.215	0.808	–
**TMT A (time)**	23.5 ± 5.5	27.4 ± 13.6	18.5 ± 4.4	*F_Q_*	0.934	0.401	–
**TMT B (time)**	62.1 ± 17.7	77.6 ± 53.1	52.4 ± 23.7	*F_Q_*	0.245	0.783	–
**PVF**	25.9 ± 10.5	24.1 ± 10.9	35.3 ± 11.7	*F_Q_*	1.925	0.158	–
**SVF**	73.9 ± 20.2	72.7 ± 15.5	90.7 ± 13.4	*F*	2.702	0.079	–

Mean ± Standard deviation.

F = ANCOVA test, *F_Q_* = Quade nonparametric ANCOVA test.

a Bonferroni correction for multiple comparison was used.

*p < 0.05.

WCST, Wisconsin Card Sorting Test-64; TMT, Trail Making test; PVF, Phonemic Verbal fluency task P-R-M; SVF, Semantic Verbal fluency task.

Possible ranges of scores: Digits Forward subtest = [0, 16]; Digits Backward subtest = [0, 14]; Letter-number sequencing = [0, 21]; Stroop Word Test = [0; ∞[; Stroop Color Test = [0; ∞[; Stroop Word-Color Test = [0; ∞[; WCST correct responses = [0, 64]; WCST total errors = [0, 64]; WCST perseverative responses = [0, 63]; WCST categories = [0, 6]; TMT-A (time, seconds) = [0, ∞[; TMT-B (time, seconds) = [0; ∞[; PVF =[0; ∞[; SVF = [0; ∞[.

*Working memory.* ANCOVA analysis showed statistically significant differences in the performance of Digits Forward subtest, with worse working memory performance in ASD group than the SCZ group (7.2 ± 2.1 vs. 9.3 ± 1.9, p = 0.003; Cohen’s d: 1.05). On Digits Backward subtest and Letter-number sequencing, there were no significant differences between groups.

*Inhibitory control.* For this EF component, we found a significant group difference in the Stroop Word Test, and statistical analysis showed that the ASD group was slower than the control group (77.7± 17.9 vs. 98.0 ± 12.7, *p* = .009; Cohen’s *d*: 1.31).

*Planning and Set-shifting ability*. ANCOVA analysis showed that the three groups performed similarly in the Wisconsin Card Sorting Test, and in the Trail Making Test A and B.

*Verbal fluency.* Results did not indicate significant group differences in the performance of Phonemic Verbal fluency task P-R-M, and Semantic Verbal fluency task.

**Figure 1:**
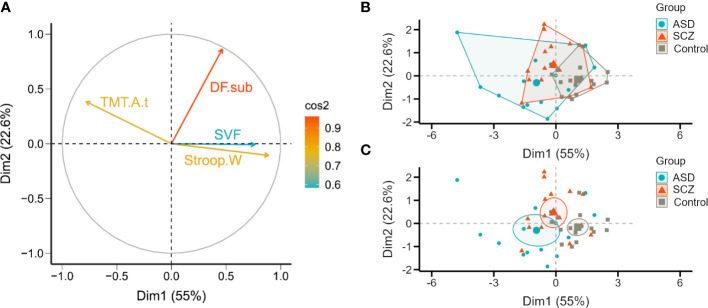
2D Principal component analysis (2D-PCA) of all 45 participants from the three cohorts, schizophrenia (SCZ), autism spectrum disorder (ASD) and controls based on normalized neuropsychological tests’ scores for executive function. **(A)** The variables under analysis are represented in a correlation circle over the space formed by the two principal components. The length of the vector reflects the strength of the correlation with each dimension and the color, the overall contribution of the variable (based on the cosine squared). **(B, C)** Participants are represented in the biplots and marked according to the group, SCZ (orange triangles), ASD (blue circles), and control (grey squares). The boundaries overlaid around group means (larger markers) represent in **(B)** a convex hull of the set of points of each group and in **(C)** the 95% confidence ellipses. DF.sub, Digits Forward subtest; Stroop.W, Stroop Word Test; TMT.A.t, Trail Making Test A (time); SVF, Semantic Verbal fluency task.

In PCA, two principal components were extracted from four initial variables, together explaining nearly 77.6% of the total variation. On average, the first and second components explained 55% ± 1.48 and 22.6% ± 0.95 of the variance. The variables that contributed most to these two components altogether were the Digits Forward subtest and the Stroop Word Test whereas the Semantic Verbal fluency task was the lowest contributor (**Figure 1A**). The PCA of the standardized metrics of the EF neuropsychological tests displayed a substantial overlap between the three groups (**Figure 1B**), especially between the two clinical groups, SCZ and ASD (**Figure 1C**).

## 4 Discussion

We compared four subdomains of EF: working memory, inhibition, planning and set-shifting, and verbal fluency, in adults with SCZ, ASD, and controls matched for age, sex, education level, and handedness. More specifically, we asked if distinct executive dysfunction patterns could be found between SCZ and ASD.

There is mounting interest in investigating the EF profile of adults with SCZ and ASD, to answer core questions about the unique aspects of both disorders. Noteworthy, SCZ and ASD are neurodevelopmental disorders, in which several studies have documented a high genetic, neuroimaging, and clinical overlap. Differential diagnosis is often challenging, namely because ASD without intellectual disability is often only diagnosed in early adulthood, which can be hard to differentiate from SCZ due to common clinical presentations, especially when manifestations include impairments in social interaction, inability to understand emotions, scarcity of psychotic symptoms or prominence of obsessive-compulsive symptoms. Also, SCZ is usually diagnosed at early stages of life.

Overall, we found no major differences in EF tests’ performance, particularly between the clinical groups, as assessed by a comprehensive battery of neuropsychological tests, where Performance-IQ was considered as covariate. It is important to note that distinct sampling strategies, with significant homogeneity in our case, may have particular impact in neuropsychological evaluation studies, which might influence the detection of EF differences. Nevertheless, in our cohort, the ASD group was significantly impaired on the Digits Forward subtest compared to the SCZ group, suggesting a particular deficit in *working memory* in ASD. The ASD group also showed slower response times on the Stroop Word-Color Test than the control group, reflecting an impairment in *inhibitory control*, and these results are in line with a recent meta-analysis (36).

### Executive function comparisons between schizophrenia and autism spectrum disorder

Two separate (not allowing for direct comparison) meta-analyses of EF in SCZ (37) and in ASD (36) have shown impaired performance in EF in both conditions. In SCZ, the meta-analysis showed significantly impaired performance in all subtests of the EF neuropsychological battery (Behavioural Assessment of Dysexecutive Syndrome – BADS), with a very large effect size in complex forward planning, inhibition, cognitive flexibility, and novel problem solving (37). In ASD, the other meta-analysis showed significantly reduced performance in a set of neuropsychological tests (Behavior Rating Inventory Task – BRIEF, Luria hand game, Stroop Test, Card Sorting Task, and multiple others) in subdomains of EF: planning, working memory, inhibition, and flexibility (36). Also, other studies (38) found executive dysfunction which persisted across development in the ASD group, in comparison with neurotypical controls. However, no differential changes were found across EF subdomains (working memory, response inhibition, planning, fluency, mental flexibility, and concept formation), reflecting an overall and not fractionated impairment in EF performance. The still unclear scenario regarding differential dysfunction in EF subdomains in ASD is in accordance with previous research focusing on aberrant brain connectivity in predicting cognitive deficits and symptom severity in ASD (39) and is due to the scarcity of direct comparison studies.

We found no differences between SCZ and ASD groups regarding three EF subdomains: inhibitory control, planning and set-shifting ability, and verbal fluency suggesting a similar performance in these subdomains. The available literature addressing explicit comparisons shows heterogeneous findings. While some studies (9, 11) reported that patients with SCZ performed worse than ASD in processing speed or in updating and shifting (12); another study did not find any significant differences in EF subdomains between SCZ and ASD (10).

Importantly, as a potential explanation for existing discrepancies, in SCZ adults EF performance depends on moderator variables such as medication, symptoms level, duration of disease, age, sex, education, or general cognition (37). In our SCZ cohort, symptoms level was measured with PANSS, namely negative symptoms with PANSS-N (16.6 ± 7.1, range score 7 - 49) that can influence EF performance. Moreover, we focused on young, all male for sample homogeneity, with education level similar to other groups, while intelligence was evaluated and taken into account. Antipsychotic medication was at the minimum dose and measured with CPZE, although a meta-analysis on cognitive performance in drug-naïve SCZ reported that significant cognitive impairments are evident even at an early stage of SCZ in unmedicated patients (40).

In ASD it is harder to have an accurate clinical identification, due to the inherently difficult diagnostic approach, as a result of being part of a spectrum, which may lead to greater cognitive variability (9). Individuals with ASD continue to develop skills throughout their life, which may help to compensate for their impairments (41). Also, ASD individuals self-report more difficulties than SCZ individuals related to EF and adaptive behaviors in everyday life situations (42). Future research should focus more on combination types of assessment such as neuropsychological, and more ecologically valid evaluations of EF.

Two principal components were able to explain nearly 77.6% of the variance in the data. The larger contributors were Digits Forward subtest and Stroop Word Test, evaluating *working memory* and *inhibitory control* subdimensions, respectively. Semantic Verbal fluency task was the least important variable.

These dimensions are relevant as set-shifting, and inhibition are considered “higher-order” EF whereby an individual needs to rely on multiple executive capacities to successfully simulate and evaluate a sequence of events prior to their execution (43).

### Limitations

There are some limitations of this study to be considered. First, the relatively small-sized sample which was traded-off with the search for homogeneity. However, some possible confounders such as age, sex, laterality, and educational level were considered, increasing sample homogeneity. Second, we assessed and controlled current medication use, namely antipsychotics, but its possible effect on EF measures in SCZ group cannot be entirely ruled out. Third, we have not included females in our sample that may have different EF performances, because significant sex differences in EF, explained by different strategies employed, supported by different circuit and/or neurochemical mechanisms are utilized by males and females to solve the same cognitive problems (44). Fourth, we only analyzed EF performance, therefore, future studies should explore the relationship between EF performance and functioning using PSP. Our results cannot be generalized to non-verbal individuals with ASD and with comorbid intellectual disability, and it is not clear whether the same conclusions can be drawn for SCZ group with florid symptoms and treatment resistant SCZ.

### Discrimination of low and high-functioning groups may help to identify those eligible for cognitive rehabilitation

To sum up, adults with SCZ and ASD matched for age, sex, education level, and handedness share executive dysfunction patterns, as assessed by neuropsychological testing, while dimensional analysis suggested differences in level of functioning that may help select patients for tailored cognitive interventions.

Future research should directly study the relationship between executive (dys)function and behavioral manifestations. Clinicians should be aware that individuals in this situation try to compensate in daily life executive dysfunction but may suffer its social consequences. The EF measures that mostly explained the variance in the sample were related to *working memory* and *inhibition*. This suggests the need for patient stratification across conditions for cognitive intervention.

## Data availability statement

The raw data supporting the conclusions of this article will be made available by the authors, without undue reservation.

## Ethics statement

The studies involving humans were approved by the local Ethics Committees of the Faculty of Medicine of the University of Coimbra (ref. CE-043/2020) and Coimbra Hospital and University Centre (ref. CHUC-109-18) in accordance with the Declaration of Helsinki. The participants provided their written informed consent to participate in this study.

## Author contributions

SMo: Conceptualization, Formal analysis, Investigation, Visualization, Data curation, Writing – original draft. OCd'A: Formal analysis, Investigation, Methodology, Writing – review & editing. SC: Investigation, Methodology, Visualization, Validation, Writing – review & editing. SMe: Investigation, Methodology, Validation, Writing – review & editing. GA: Investigation, Methodology, Validation, Writing – review & editing. VG: Investigation, Methodology, Validation, Writing – review & editing. CB: Investigation, Methodology, Validation, Writing – review & editing. DP: Investigation, Validation, Writing – review & editing. AM: Conceptualization, Supervision, Validation, Writing – review & editing. MC-B: Conceptualization, Data curation, Funding acquisition, Investigation, Project administration, Resources, Supervision, Validation, Writing – review & editing.
