# Supplementary material for: Executive function in schizophrenia and autism in adults shares common components separating high and low performance groups
Source: Front Psychiatry. 2024 Apr 18;15:1381526. doi: 10.3389/fpsyt.2024.1381526 (PMC11064061; doi:10.3389/fpsyt.2024.1381526)
Supplement: Supplementary file 1 [file Image_1.pdf]

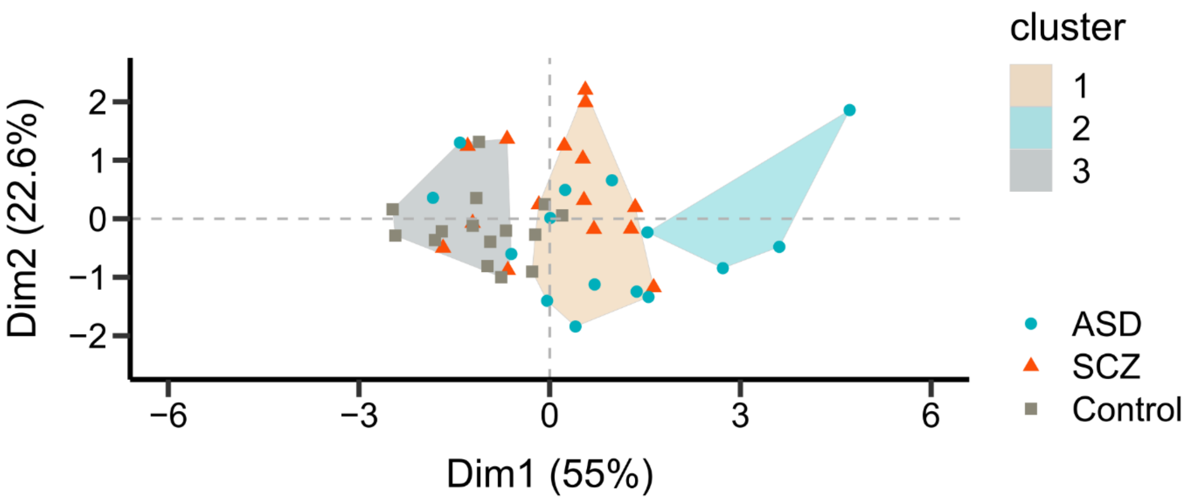

Figure S1. An exploratory unsupervised k-means cluster analysis was performed on the same (Digits Forward subtest, Stroop Word Test, Trail Making Test A (time), Semantic Verbal fluency task) neuropsychological tests' scores (normalized to Z scores) for executive function. K-means clustering assumed 3 clusters and 25 random starting assignments. Three well defined clusters (convex hull) were defined: 1) an intermediate (orange), more heterogeneous (n=22) but with higher proportion of individuals from the clinical groups (81.8%); 2) on the right (blue), constituted exclusively by autism spectrum disorder (ASD, blue circles) participants (n=4); 3) on the left, mixed (n=19), but especially including controls (grey squares, 57.9%). Data analysis was conducted using the '*factoextra'* package in the R Studio software (version 4.2.1). SCZ = schizophrenia (orange triangles).
